# Neoxanthin: A Promising Medicinal and Nutritional Carotenoid

**DOI:** 10.3390/md23080317

**Published:** 2025-08-01

**Authors:** Jiarong Zhao, Gengjie Zhuang, Jinrong Zhang

**Affiliations:** 1 College of Food Sciences and Engineering, Ningbo University, Ningbo 315211, China; zhaojiarong0131@163.com; 2 School of Marine Sciences, Ningbo University, Ningbo 315211, China; zhuanggengjie123@163.com

**Keywords:** source, biosynthetic pathways, extraction, pharmacological activities, microalgae

## Abstract

Neoxanthin is a xanthophyll carotenoid with high-value nutritional functions for human health due to its anti-cancer, anti-oxidative, and anti-obesity activities. In this present work, we systematically reviewed the structure, source, and biosynthetic pathways of neoxanthin, and discussed the advantages and disadvantages of the prevailing extraction methods of neoxanthin. Meanwhile, this review described the latest research progress on the pharmacological activities of neoxanthin. Finally, we concluded with a discussion on the main challenges of neoxanthin production from microalgae, and proposed some future development prospects and potential solutions.

## 1. Introduction

Carotenoids are a class of natural isoprene pigments with high-value nutritional functions for human health [1]. The study of carotenoids has a history of around 200 years [2]. The earliest record about the presence of natural carotenoid was published in 1831, when Professor Heinrich Wilhelm Ferdinand Wackenroder discovered the existence of natural carotenoid in carrot juice and its benefits [3]. In 1929, Professor Harry Steenbock proposed the possibility about a relationship between vitamin A activity and carotenoid pigmentation [4]. The possibility has caused great attention to natural carotenoids and their biological activities. In 1931, the chemical structure of *β*-carotene was described in detail by Professor Paul Karrer, who won the Nobel Prize in Chemistry due to his discovery [5]. Currently, the established carotenoids database could provide the structure, chemical fingerprints, distribution, and source information on 1204 natural carotenoids, as well as their biological functions (See: http://carotenoiddb.jp, accessed on 29 July 2025). Carotenoids have been widely applied in food, pharmaceutics, and cosmetics fields [6,7]. At present, some natural carotenoids, mainly including astaxanthin, *β*-carotene, fucoxanthin, lutein, lycopene, and zeaxanthin, are the main carotenoids possessing high added value with the largest market share [8].

Neoxanthin, a C40 epoxycarotenoid, has some remarkable bioactivities, including anti-cancer and anti-bacterial (See: http://carotenoiddb.jp, accessed on 29 July 2025). Currently, neoxanthin has been found in a variety of high plants, algae, and animals. In diverse high plants and algae, neoxanthin is one of the key photosynthetic xanthophylls [9], Notably, many microalgae are regarded as potential source species due to their rich content of neoxanthin, especially diverse microalgae species of the *Chlorophyta phylum* and *Chlorophyta* class, including *Scenedesmus obliquus* [10], *Chlorella vulgaris* [11], and *Chaetomorpha antennia* [12]. Neoxanthin has some remarkable bioactivities, including anti-cancer, anti-oxidant, and anti-obesity [13]. Therefore, neoxanthin is regarded as a high-added-value carotenoid from high plants and microalgae, which has significant potential for applications in the food, pharmaceutical, and cosmetic industries [14].

In this paper, we present a systematic review of neoxanthin focusing on its source, biosynthetic pathways, prevailing extraction methods, and biological activities. Furthermore, the review discussed the challenges and opportunities facing the future research field of neoxanthin.

## 2. Structure of Neoxanthin

**Figure 1:**
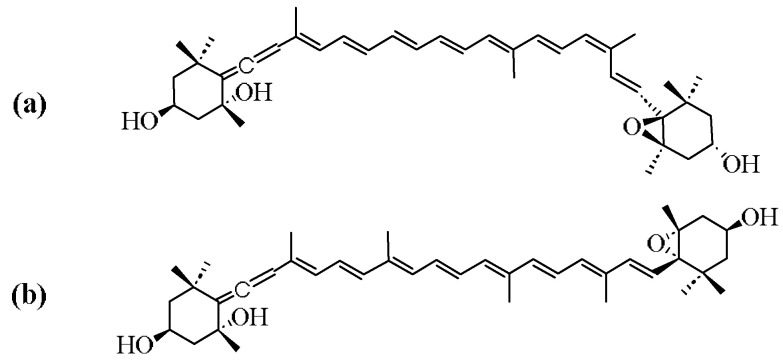
Structure of (**a**) 9′-*cis* neoxanthin (**b**) all-*trans* neoxanthin.

The study of neoxanthin has a history of around 100 years. The earliest report was in 1938, when Professor Strain first discovered the presence of neoxanthin and named it, which was a common carotenoid distributed in barley leaves. Together with lutein and violaxanthin, neoxanthin is regarded as one of the three major xanthophylls of leaves [15,16]. As a potent light-harvesting carotenoid, neoxanthin is a major xanthophyll carotenoid in dark green leafy vegetables [17], additionally, it is a precursor of the plant hormone abscisic acid in these vegetables [18]. The molecular formula of neoxanthin is C_40_H_56_O_4_, with a molecular weight of 600.87 Da. It is insoluble in water but soluble in organic solvents such as ethanol, methanol, and acetone [19]. The structure of neoxanthin contains an unusual allenic bond, 8 conjugated double bonds, some oxygenic functional groups, including epoxy and hydroxyl moieties, and other functional groups [20]. Like other carotenoids, neoxanthin is chemically unstable, which makes it sensitive to heat and light that can impair its structure due to the conjugated polyenes in its chemical structure [21] (Figure 1).

In nature, neoxanthin mainly exists in two forms of geometrical isomer: all-*trans* neoxanthin and 9′-*cis* neoxanthin [22]. Their chemical structures are shown in Figure 1. The two isomers can be distinguished using ultraviolet absorption spectra. In ethanol, all-*trans* neoxanthin presents the main absorption peaks at 418, 442, and 471 nm [23]. But the 9′-*cis* neoxanthin presents the main absorption peaks at 413, 437, and 466 nm [24]. In higher plants, two forms of neoxanthin are found in different organs. 9′-*cis* neoxanthin is found in leaves, which can accomplish photosynthesis, while all-*trans* neoxanthin is found in some organs like petals and fruits that cannot carry out photosynthesis [18]. As far as algae are concerned, 9′-*cis* neoxanthin is found in nearly all microalgae species of the *Chlorophyta phylum* [10]. However, all-*trans* neoxanthin is the characteristic isomer found in microalgae species of *Mesostigmatophyceae* [25].

## 3. Neoxanthin Source

**Figure 2:**
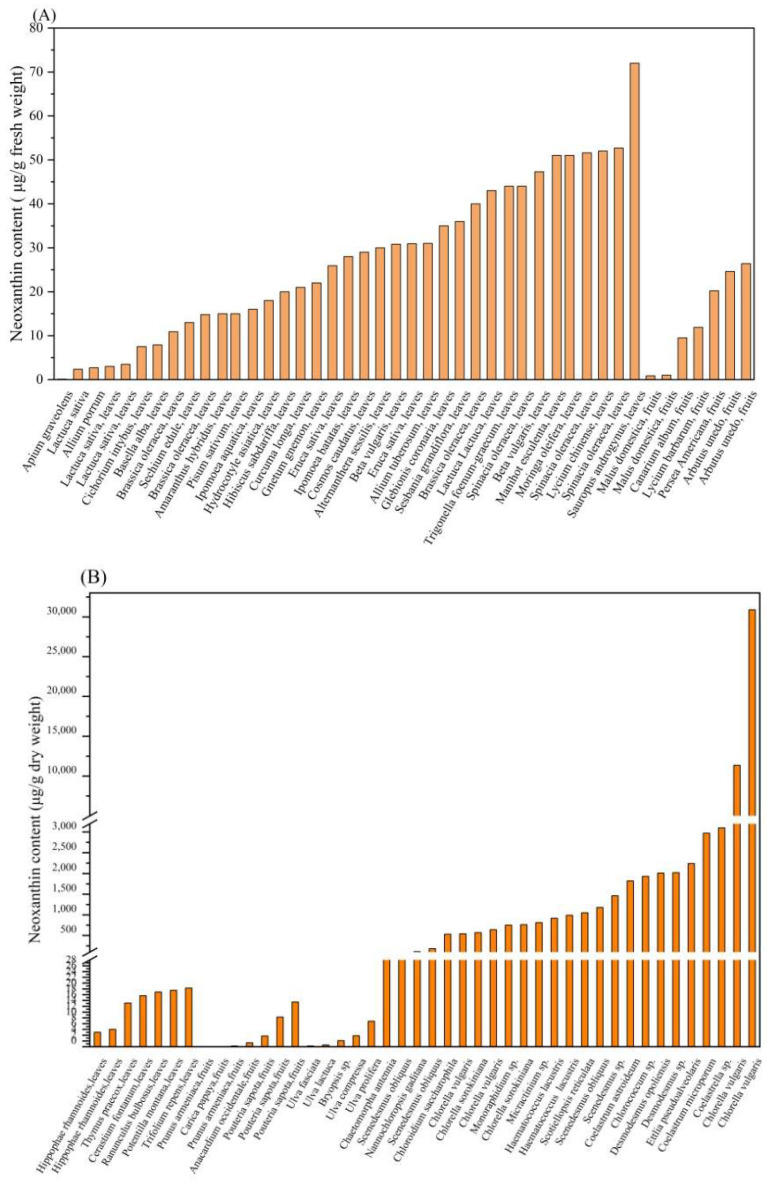
Neoxanthin content in various sources. (**A**) Neoxanthin content expressed in terms of fresh weight (FW) of high plants; (**B**) Neoxanthin content expressed in terms of dry weight (DW) of high plants and microalgal species.

Neoxanthin has been found in a variety of higher plants and microalgae. High plants are one of the main sources of neoxanthin [9]. The neoxanthin content in the leaves and fruits of the high plants is shown in Table 1 and Table 2, while that in microalgal species is shown in Table 3. Additionally, the neoxanthin content in high plants and microalgal species is displayed in Figure 2.

**Table 1:** Neoxanthin content in the leaves of high plants.

High Plant	Content of Neoxanthin	Reference
*Allium porrum*	2.7 ± 0.9 ^a^	[26]
*Allium tuberosum*	31 ± 2 ^a^	[26]
*Alternanthera sessilis*	30 ± 3 ^a^	[26]
*Amaranthus hybridus*	15 ± 5 ^a^	[26]
*Apium graveolens*	0.11 ± 0.09 ^a^	[26]
*Basella alba*	7.9 ± 0.7 ^a^	[26]
*Beta vulgaris*	30.8 ^a^	[27]
*Beta vulgaris*	47.3 ^a^	[27]
*Brassica oleracea*	40 ± 10 ^a^	[26]
*Brassica oleracea*	14.8 ^a^	[28]
*Brassica oleracea*	10.9 ^a^	[28]
*Cerastium fontanum*	17.6 ± 0.860 ^b^	[29]
*Cichorium intybus*	7.5 ^a^	[28]
*Cosmos caudatus*	29 ± 7 ^a^	[26]
*Curcuma longa*	21 ± 1 ^a^	[26]
*Eruca sativa*	30.9 ^a^	[27]
*Eruca sativa*	25.9 ^a^	[27]
*Glebionis coronaria*	35 ± 6 ^a^	[26]
*Gnetum gnemon*	22 ± 2 ^a^	[26]
*Hibiscus sabdariffa*	20 ± 2 ^a^	[26]
*Hippophae rhamnoides*	5.0 ± 0.0 ^b^	[30]
*Hippophae rhamnoides*	6.0 ± 0.0 ^b^	[30]
*Hydrocotyle asiatica*	18 ± 1 ^a^	[26]
*Ipomoea aquatica*	16 ± 1 ^a^	[26]
*Ipomoea batatas*	28 ± 3 ^a^	[26]
*Lactuca sativa* var. *capitata*	3 ± 1 ^a^	[26]
*Lactuca sativa* var. *Longifolia*	43 ± 1 ^a^	[26]
*Lactuca sativa* var. *Longifolia*	2.36 ^a^	[27]
*Lactuca sativa* var. *Longifolia*	3.49 ^a^	[27]
*Lycium chinense*	52 ± 8 ^a^	[26]
*Manihot esculenta*	51 ± 5 ^a^	[26]
*Moringa oleifera*	51 ± 12 ^a^	[26]
*Pisum sativum*	15 ± 2 ^a^	[26]
*Potentilla montana*	19.5 ± 0.787 ^b^	[29]
*Ranunculus bulbosus*	18.9 ± 0.179 ^b^	[29]
*Sauropus androgynus*	72 ± 6 ^a^	[26]
*Sesbania grandiflora*	36 ± 4 ^a^	[26]
*Sechium edule*	13 ± 6 ^a^	[26]
*Spinacia oleracea*	44 ± 9 ^a^	[26]
*Spinacia oleracea*	52.7 ^a^	[27]
*Spinacia oleracea*	51.6 ^a^	[27]
*Thymus praecox*	15.1 ± 0.0803 ^b^	[29]
*Trifolium repens*	20.3 ± 0.556 ^b^	[29]
*Trigonella foenum-graecum*	44 ± 7 ^a^	[26]

^a^ Neoxanthin content profile expressed as μg/g fresh weight (FW) of high plants. ^b^ Neoxanthin content profile expressed as μg/g dry weight (DW) of high plants.

The experimental studies have demonstrated that the neoxanthin content in dry leaves of high plants is about 5.00~20.30 μg/g dry weight [29], while that in the fresh leaves is about 0.11~72.00 μg/g fresh weight [26].

**Table 2:** Neoxanthin content in the fruit of high plants.

High Plant	Content of Neoxanthin	Reference
*Arbutus unedo*	26.4 ± 1.8 ^a^	[31]
*Arbutus unedo*	24.6 ± 1.9 ^a^	[31]
*Anacardium occidentale*	1.36 ^b^	[32]
*Canarium album*	9.5 ^a^	[33]
*Carica papaya*	0.007 ^b^	[34]
*Lycium barbarum*	11.9 ± 0.0 ^a^	[35]
*Malus domestica*	0.99 ± 0.05 ^a^	[36]
*Malus domestica*	0.87 ± 0.05 ^a^	[36]
*Persea Americana*	20.2 ± 2.80 ^a^	[37]
*Pouteria* *sapota*	15.45 ± 1.32 ^b^	[38]
*Pouteria* *sapota*	10.24 ± 2.63 ^b^	[38]
*Pouteria* *sapota*	3.70 ± 0.99 ^b^	[38]
*Prunus armeniaca*	0.005 ^b^	[39]
*Prunus armeniaca*	0.257 ^b^	[39]

^a^ Neoxanthin content profile expressed as μg/g dry weight (DW) of high plants. ^b^ Neoxanthin content profile expressed as μg/g fresh weight (FW) of high plants.

In addition, the neoxanthin content in dry fruit of high plants is about 0.02~51.00 μg/g dry weight [31], while that in the fresh fruit is about 0.005~15.45 μg/g fresh weight [38]. Interestingly, two forms of neoxanthin (all-*trans* neoxanthin and 9′-*cis* neoxanthin) are found in the fruits of two high plants, *Arbutus unedo* and *Malus domestica* [31,36]. In terms of a promising source of neoxanthin, high plants are difficult to be a competitive neoxanthin feedstock due to some disadvantages, including their low neoxanthin content, and their limited growth dependent on the season [40]. Notably, the content of neoxanthin in the microalgae is about 0.26~30,880 μg/g dry weight [11,12].

**Table 3:** Neoxanthin content in Chlorophyta microalgal species.

Microalgal Species	Neoxanthin Content	Reference
*Bryopsis* sp.	2.11 ± 0.10 ^a^	[12]
*Chaetomorpha antennia*	33.35 ± 0.23 ^a^	[12]
*Chloroidium saccharophilum (formerly Chlorella saccharophila)*	530 ± 40 ^a^	[10]
*Chlorella sorokiniana*	570 ± 20 ^a^	[10]
*Chlorella sorokiniana*	760 ± 120 ^a^	[10]
*Chlorella vulgaris*	540 ± 80 ^a^	[10]
*Chlorella vulgaris*	640 ± 50 ^a^	[10]
*Chlorella vulgaris*	11,350 ± 17 ^a^	[11]
*Chlorella vulgaris*	30,880 ± 426 ^a^	[11]
*Chlorococcum* sp.	1930 ± 130 ^a^	[10]
*Coelastrella* sp.	3100 ± 220 ^a^	[10]
*Coelastrum astroideum*	1820 ± 80 ^a^	[10]
*Coelastrum microporum*	2970 ± 170 ^a^	[10]
*Desmodesmus opoliensis*	2010 ± 160 ^a^	[10]
*Desmodesmus* sp.	990 ± 70 ^a^	[10]
*Desmodesmus* sp.	2020 ± 150 ^a^	[10]
*Ettlia pseudoalveolaris*	2240 ± 90 ^a^	[10]
*Haematococcus lacustris (formerly Haematococcus pluvialis)*	920 ± 20 ^a^	[10]
*Micractinium* sp.	810 ± 50 ^a^	[10]
*Monoraphidium* sp.	750 ± 70 ^a^	[10]
*Nannochloropsis gaditana*	110 ± 20 ^a^	[41]
*Scenedesmus obliquus*	1180 ± 30 ^a^	[10]
*Scenedesmus obliquus*	55.72 ± 1.72 ^a^	[42]
*Scenedesmus obliquus*	180.33 ± 11.23 ^a^	[42]
*Scenedesmus* sp.	1460 ± 100 ^a^	[10]
*Scotiellopsis reticulata*	1050 ± 110 ^a^	[10]
*Ulva compressa*	3.81 ± 0.08 ^a^	[12]
*Ulva fasciata*	0.26 ± 0.00 ^a^	[12]
*Ulva lactuca*	0.61 ± 0.07 ^a^	[12]
*Ulva prolifera*	8.84 ± 0.12 ^a^	[12]

^a^ Neoxanthin content profile expressed as μg/g dry weight (DW) of microalgal species.

Among the published reports, the highest recorded neoxanthin content in the microalga *Chlorella vulgaris* is 30880 μg/g dry weight [11]. Thus, the bioproduction of neoxanthin by microalgae is considered a sustainable and promising option [43]. Generally, microalgae are considered a promising source for diverse carotenoids, such as astaxanthin and fucoxanthin [44]. Microalgae are regarded as an excellent source due to some advantages, including the diversity of microalgal species, their rapid growth rate, the production of target carotenoid under controlled culture conditions, and high content of carotenoid content [45,46]. In conclusion, the bioproduction of neoxanthin is considered a promising and sustainable option. 

## 4. Biosynthetic Pathway of Neoxanthin

**Figure 3:**
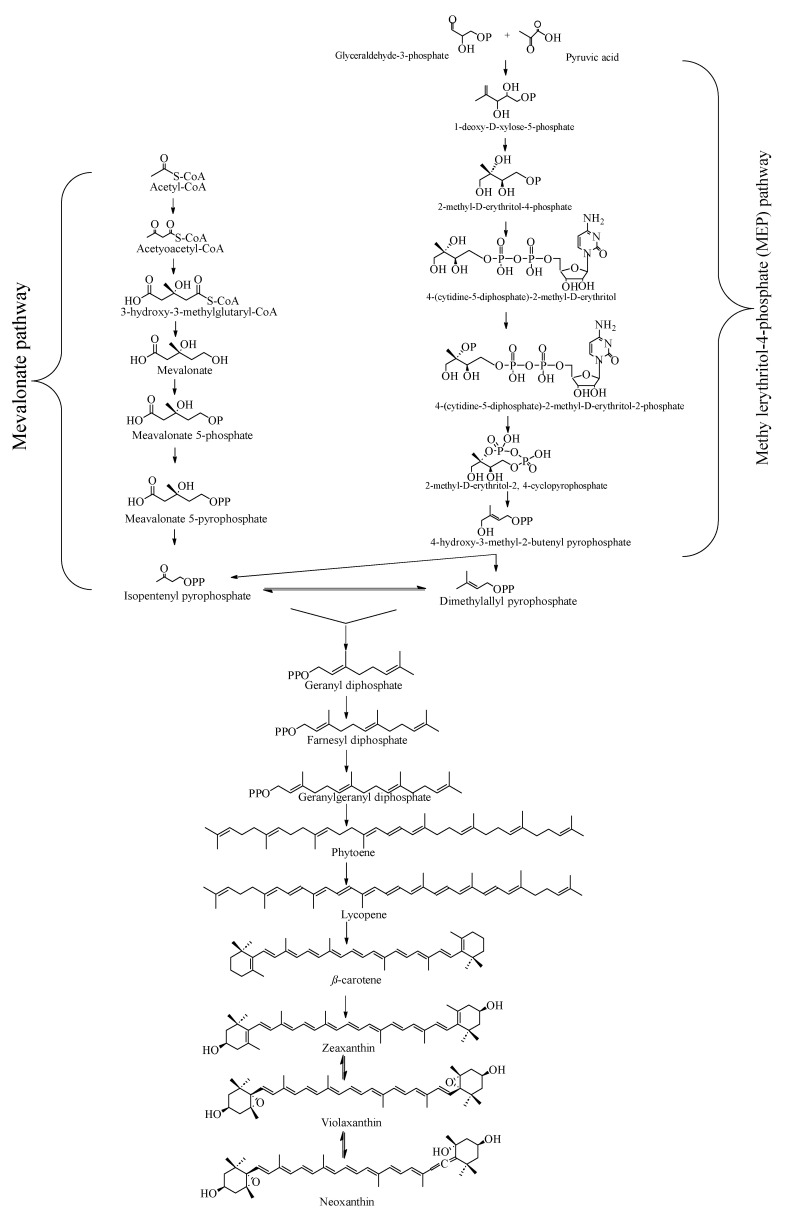
Biosynthetic pathway of neoxanthin.

The biosynthetic pathway of neoxanthin is shown in Figure 3. Mevalonate (MVA) pathway mainly occurs in the cytoplasms of some organism including eukaryotes, archaea and plants, while methyl erythritol-4-phosphate (MEP) pathway mainly occurs in the chloroplasts of organisms such as bacteria and plants. Isopentenyl pyrophosphate (IPP) and dimethylallyl pyrophosphate (DMPP) are the precursor metabolites of carotenoid biosynthesis, which are synthesized by the mevalonate (MVA) pathway and the methylerythritol 4-phosphate (MEP) pathway, respectively [47]. Then, Dimethylallyl pyrophosphate(DMAPP) and IPP combine via a three-step condensation reaction to produce geranylgeranyl diphosphate (GGPP), which is the immediate precursor of carotenoid synthesis. Two GGPP molecules are then condensed by the enzymatic action of phytoene synthase (PSY) to produce phytoene (C40) [48,49]. Then phytoene is converted to lycopene by a multi-step process through sequential desaturation and isomerization reactions. Thereafter, lycopene is converted to *β*-carotene, which is one of key precursors of xanthophyll compounds. *β*-carotene is converted to zeaxanthin by double hydroxylation reactions catalyzed by *β*-carotene hydroxylase (BCH). Then zeaxanthin is converted to violaxanthin through two epoxidation reactions in the presence of the zeaxanthin epoxidase (ZEP). Finally, violaxanthin is converted to neoxanthin via the enzyme neoxanthin synthase (NXS) [50,51,52].

## 5. Extraction of Neoxanthin

Currently, some methods have been explored for the extraction of neoxanthin, including organic solvents extraction [42,53], ionic liquids extraction [54,55], supercritical liquid extraction [56], ultrasound-assisted extraction [57,58], and pressurized liquid extraction [11] (see Table 4).

### 5.1. Organic Solvents Extraction

Neoxanthin, a lipid-soluble xanthophyll with relatively weak polarity, is easily soluble in polar organic solvents [59]. Generally, some organic solvents, including dimethyl ether, acetone, and ethanol, have been used for the extraction of neoxanthin [60]. Notably, a pioneering study has highlighted the green extraction of liquefied dimethyl ether (DME), which was an efficient solvent for the extraction of neoxanthin from the microalga *Chlorococcum humicola* [53]. The optimal extraction conditions were determined as follows: liquefied dimethyl ether, 41 °C, 20 min, a liquid-to-solid ratio of 45:1 (*w*/*w*), and once extraction process. Under these conditions, the yield of neoxanthin reaches 2.55 mg/g wet sample, and the recovery rate of neoxanthin is 95%. As a medium polarity solvent, liquefied dimethyl ether is regarded as an alternative green solvent for the neoxanthin extraction due to its some advantages, including partial miscibility with water and a low normal boiling point (−24 °C) [61,62].

Generally, organic solvents extraction is a traditional and common technology that has been widely used for the extraction of diverse compounds on a commercial scale due to its advantages [63], including simple operation, wide application, good industrial manufacturing equipment, and rich manufacturing experience on a commercial scale [64]. However, this method possesses some disadvantages, including the toxicity of some organic solvents, high solvent consumption, low selectivity of extraction, and low extraction efficiency [65]. Thus, ethanol is regarded as a suitable solvent for extraction due to its good safety and low cost [66,67]. Ethanol has been approved for the extraction of multiple compounds on a commercial scale [68].

### 5.2. Ionic Liquids Extraction

In terms of neoxanthin extraction by using ionic liquids technology, some studies have been carried out on diverse microalgae, including *Caryocar Brasiliense* [55], *Chlorella sorokiniana* [54], and *Scenedesmus obliquus* [42]. Generally, ionic liquid is a mixture composed of ionic liquids and/or organic solvents, including 1-hexyl-3-methylimidazolium chloride ([HMIM][Cl]), 1-butyl-3-methylimidazolium tetrafluoroborate ([BMIM][BF4]), ethanol, and chloroform [69]. Interestingly, an essential study has highlighted the extraction of the mixture ([BMIM][BF4]:ethanol = 1:3), which was an efficient ionic liquid solvent for neoxanthin extraction from the microalga *Scenedesmus obliquus* [42]. The optimum extraction conditions were determined as follows: the mixture ([BMIM][BF4]:ethanol = 1:3), 3 min, a liquid-to-solid ratio of 10:1 (mL/g), and extraction three times. Under the conditions, the yield of neoxanthin reaches 122.66 μg/g dry sample.

Presently, ionic liquids extraction has been used for the extraction of diverse compounds, including neoxanthin, erythromycin A, and astaxanthin [70]. As a new type of green solvents, multiple ionic liquids (ILs) possess some advantages, including high thermal stability, high conductivity, low vapor pressure, and an extensive range of electrochemical properties [71,72]. Generally, ionic liquids (ILs) are regarded as promising sustainable extraction solvents due to their high extraction efficiency, good biocompatibility with multiple compounds, low cost, and low environmental impact [73].

### 5.3. Supercritical Liquid Extraction

Supercritical liquid extraction has been widely applied for the extraction of diverse non-polar compounds, including neutral oils, essential oils, and various carotenoids [74]. Notably, an interesting study has highlighted the importance of supercritical liquid of a co-solvent composed of CO_2_ (CO_2_ density, 830 g L^−1^) and 10% ethanol during the neoxanthin extraction from *Scenedesmus* sp. [56]. The optimal extraction conditions were determined as follows: the supercritical liquid of a co-solvent [CO_2_ (CO_2_ density, 830 g L^−1^) and 10% ethanol], 60 °C, 60 min, pressure of 300 bar, and liquid flow of 2 mL min^−1^. Under the conditions, the yield of neoxanthin reaches 670.8 μg/g dry sample [56].

Generally, supercritical carbon dioxide (SCCO_2_) has been widely applied for multiple compounds for food application due to its environmentally friendly and high safety [75,76]. However, supercritical liquid extraction is still limited owing to its high cost. Particularly, the extraction using a co-solvent composed of CO_2_ and ethanol requires a rather high operating pressure in the range of 20~60 MPa [77,78].

### 5.4. Ultrasound-Assisted Extraction

Generally, ultrasound-assisted extraction is regarded as an efficient method for the extraction of multiple bioactive molecules, including polyphenols, carotenoids, and flavonoids [79]. Interestingly, a study aimed to develop an eco-friendly and high-efficiency method for neoxanthin extraction from *Chlorella vulgaris* was investigated using ultrasound-assisted extraction. The optimal extraction conditions were determined as follows: Ethanol, 25 °C, 25 min, a liquid-to-solid ratio of 30:1 (mL/g), and amplitude wave of 20%. Under these conditions, the yield of neoxanthin reaches 9.83 mg/g dry weight biomass [11]. Ultrasound-assisted extraction (UAE) has been widely applied for the extraction of diverse natural molecules due to its advantages, including operation simplicity, short extraction duration, low extraction temperature, and ability to retain the original structure of natural products [80,81].

### 5.5. Pressurized Liquid Extraction

In terms of neoxanthin extraction by using pressurized liquid extraction, an interesting study has been developed from the microalga *Chlorella vulgaris* [11]. Several pressurized liquid extraction conditions were assessed, including different solvents (water, ethanol, and acetone), different extraction temperatures (50 °C, 100 °C, 150 °C, and 200 °C). The green extraction conditions were determined as follows: ethanol, 50 °C, extraction pressure of 1500 psi, and extraction time of 20 min. Under these conditions, the yield of neoxanthin reaches 11.35 mg/g dry sample [11]. Pressurized liquid extraction is an efficient method because the solvent possesses strong cell-penetrating capacity under suitable temperature and high pressure during the whole extraction procedure [11,82]. In addition, the method possesses some advantages, including green solvents, short extraction duration, and auto operation [83,84].

## 6. Biological Activities of Neoxanthin

**Figure 4:**
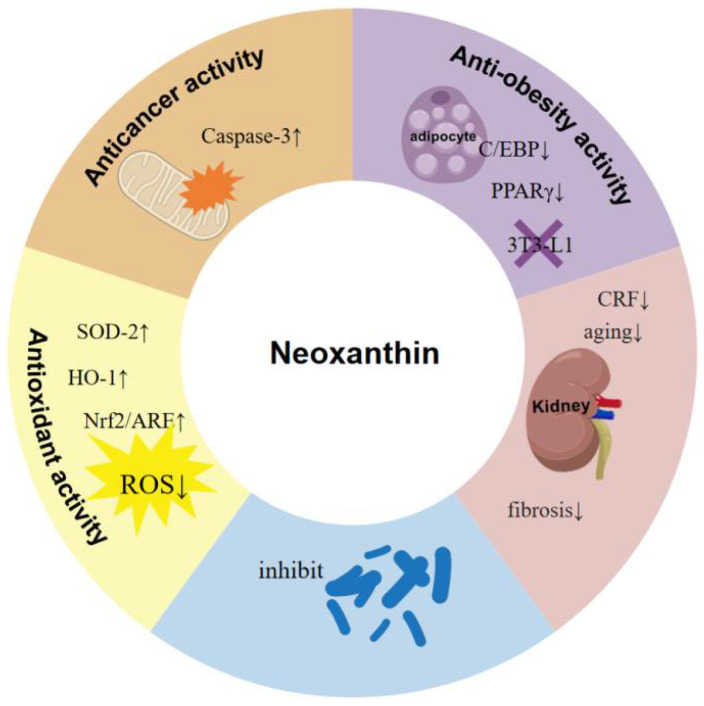
Biological activities of neoxanthin.

Carotenoids are food functional ingredients with diverse biological activities, including anti-tumor, anti-oxidant, weight loss, and anti-Alzheimer’s effects [85,86]. Some carotenoids are popular in foods, cosmetics, and health products, including astaxanthin and fucoxanthin [1,87,88]. Neoxanthin has shown diverse biological activities, including anti-tumor, anti-oxidant, and anti-obesity [13] (Figure 4).

### 6.1. Anti-Cancer Activity 

The incidence and mortality rates of cancer are increasing rapidly worldwide, which has attracted increasing attention [89]. Dietary modifications are considered one of the most promising lifestyle changes that can reduce cancer risk by around 40% [90]. Carotenoids, distributed in many fruits and vegetables, are a class of natural pigments with anti-tumor activity [91]. Neoxanthin, one of the chief carotenoids occurring in nature, has been found abundantly in diverse higher plants, such as *Spinacia oleracea*, *Citrullus vulgaris*, *Citrus sinensis*, and *Solanum lycopersicum* [9]. Regarding the anti-tumor activity of neoxanthin, it has shown a multi-target mechanism of action in anti-tumor; additionally, it significantly affects different types of tumor cells [92]. The multi-target mechanism of action of neoxanthin in multiple cancer cell lines is shown in Table 5.

The effects of 9′-*cis* neoxanthin on the induction of apoptosis in HCT116 human colon cancer cells have been assessed [93]; additionally, the underlying mechanisms for its anti-cancer effects have been elucidated. The results have shown that chromatin condensation, DNA fragmentation, and increased hypodiploid cells have been observed when the HCT116 human colon cancer cells are exposed to 20 μM neoxanthin. In addition, the treatment with neoxanthin at 5 μM significantly inhibits the proliferation of HCT116 cells. Mechanisms underlying the anti-cancer action of 9′-*cis* neoxanthin can be elucidated as follows. Neoxanthin treatment causes an enhancement in the activities of caspase-3, -8, and -9, as well as an increase in the protein levels of their active subunits, except for caspase-8. In addition, the treatment results in an early-stage reduction in mitochondrial transmembrane potential, followed by the subsequent release of cytochrome c and apoptosis-inducing factor (AIF) from the mitochondria into the cytosol. Notably, the direct exposure of neoxanthin to mitochondria isolated from HCT116 cells results in an enhanced release of cytochrome c and apoptosis-inducing factor (AIF) in a dose-dependent manner. Around 50% of the neoxanthin absorbed by the cells was observed to be accumulated within the mitochondrial fraction. These findings suggest that the accumulation of neoxanthin in the mitochondrial fraction induces a loss of mitochondrial transmembrane potential, subsequently facilitating the release of cytochrome c and AIF, thereby promoting cell apoptosis [93].

The effects of neoxanthin on the induction of apoptosis in PC-3 cells have been investigated [94], which is characterized by morphological alterations, DNA fragmentation, an elevated proportion of hypodiploid cells, and the cleavage of caspase-3 and poly (ADP-ribose) polymerase (PARP) [94]. The results suggest that the proportion of apoptotic cells exceeds 30% after the PC-3 cells are exposed to 20 μM neoxanthin for 48 h. In addition, this treatment causes a reduction in the expression levels of Bax and Bcl-2 proteins, but does not affect Bcl-XL expression. In all, neoxanthin treatment has been observed to induce apoptosis via the activation of caspase-3 in PC-3 human prostate cancer cells [94].

CagA is an oncoprotein that significantly contributes to the progression of gastric cancer through its interaction with phosphatidylserine in the host cell plasma membrane [97]. This protein represents a promising target for cancer therapy [98]. A total of 38 natural compounds from medicinal plants are screened to evaluate their effects on the interaction of CagA protein with host cell membrane phosphatidylserine (PS) through computational methods such as molecular docking and molecular dynamics simulation [99]. The results suggest that neoxanthin has strong and steady interactions between its molecules and CagA [95]. The molecular docking results indicate that neoxanthin can bind tightly to the positively charged helix α18 region (residues 610–639) of CagA protein, which is a key region for the interaction between CagA and PS. Notably, neoxanthin also exhibits the lowest binding energy (PBTOT) with −34.39 kcal/mol. The complexes of neoxanthin with CagA are the most stable; additionally, the binding free energy (PBTOT) values are the lowest. Therefore, the key pharmacophores of neoxanthin are more favorable to the binding interactions within the CagA binding site. In all, the results suggest that neoxanthin is a potentially excellent active compound against the oncoprotein CagA [95].

In oncological research, 9′-*cis* neoxanthin has demonstrated significant inhibitory effects on the viability of cervical cancer (HeLa) and lung cancer (A549) cells, with IC_50_ values of 3.8 μM and 7.5 μM, respectively [96]. The treatment with 9′-*cis* neoxanthin at 10 μM results in a significant reduction in cell viability, with values decreasing to 10.9%, 15.0%, and approximately zero for PC-3, DU 145, and LNCaP cell lines, respectively [96].

The incidence and progression of tumors are prevalent across various cancer types, including colorectal cancer, lung cancer, and others [100]. These cancers often exhibit common biochemical disruptions, such as unchecked cellular proliferation and evasion of apoptosis [101]. Neoxanthin has emerged as a potential compound for the prevention and treatment of these malignancies [102]. Several studies suggest that neoxanthin significantly impacts the proliferation of diverse tumor cell types [94]. The anti-cancer effects of neoxanthin are likely mediated through multiple pathways, including mitochondrial-induced apoptosis, reversal of multidrug resistance, caspase-3-dependent apoptosis, inhibition of oncogenic signaling, and suppression of cell proliferation [13,93,95]. Consequently, neoxanthin represents a promising candidate for the prevention and treatment of various cancers.

### 6.2. Anti-Oxidant Activity

Oxidative stress (OS) is characterized by an imbalance between the production of reactive oxygen species (ROS) and the capacity of anti-oxidant systems to neutralize these products [103]. Oxidative stress is implicated in the pathogenesis of cancer, inflammatory and hyperpigmentation disorders, neurodegenerative diseases, diabetes, and cardiovascular diseases [104]. Various carotenoids, such as astaxanthin, fucoxanthin, and lutein, are well-known for their notable anti-oxidant properties [105]. They possess the capacity to quench singlet oxygen and scavenge harmful free radicals, thereby preventing or decreasing damage to living cells [106].

Neoxanthin and lutein are purified from the microalga *Chlamydomonas reinhardtii*; additionally, their protective effects against oxidative stress-induced DNA damage have been evaluated [107]. The results show that 10.4 μM of neoxanthin inhibits about 72% of DNA damage under Fenton reaction-induced oxidative stress, while the concentration of 35 μM lutein reduces only about 55% of DNA damage. These results suggest that neoxanthin exhibits stronger anti-oxidant activity than lutein at lower concentrations, proving to be a better inhibitor of oxidatively induced DNA damage [107].

In a study investigating lipid peroxidation using mouse C3H10T1/2 fibroblasts, the anti-oxidant properties of neoxanthin were validated [13]. Uric acid and superoxide ions are generated from xanthine through the action of xanthine oxidase. Consequently, superoxide anions target the unsaturated fatty acids within the membrane, leading to the production of malondialdehyde, a critical indicator of membrane damage. It has been demonstrated that neoxanthin, like *β*-carotene, can significantly reduce malondialdehyde formation, exhibiting an activity level one hundred times greater than that of *β*-carotene [13]. In a separate investigation utilizing HepG2 hepatocyte cells, the cytotoxic effects of H_2_O_2_ and the cytoprotective efficacy of neoxanthin are assessed. Compared to *β*-carotene and lutein, pretreatment with neoxanthin offers superior protection against cell death induced by H_2_O_2_. Neoxanthin achieves this by upregulating intracellular anti-oxidant enzymes, such as HO-1 and SOD-2, enhancing the expression of anti-apoptotic proteins, and decreasing the expression of pro-apoptotic proteins [13].

In a study examining the cytotoxic effects of H_2_O_2_ on the HepG2 cell model, neoxanthin demonstrates a significant protective effect against H_2_O_2_-induced oxidative stress in HepG2 cells [108] (see Table 6). In comparison to *β*-carotene and lutein, neoxanthin pretreatment markedly enhances cell survival rates of HepG2 cells under H_2_O_2_ exposure and demonstrates more potent cytoprotective effects at lower concentrations (0.05 μM and 0.1 μM). The findings indicate that following a 4 h exposure to 0.5 mM H_2_O_2_, the cell viability in the neoxanthin pretreatment groups (0.05 μM and 0.1 μM) is significantly greater compared to the group subjected solely to H_2_O_2_ treatment. These results suggest that neoxanthin effectively mitigates H_2_O_2_-induced cytotoxicity by activating endogenous anti-oxidant signaling pathways within the cells, thereby preserving cell viability [108].

Additionally, neoxanthin can also reduce oxidative stress by directly scavenging ROS produced within cells [109]. Experimental data indicate that treatment with H_2_O_2_ significantly elevates the levels of reactive oxygen species (ROS) in HepG2 cells, with an increase of 38% compared to the control group. In contrast, pretreatment with neoxanthin at concentrations of 0.05 μM and 0.1 μM results in a reduction In ROS production by 12% and 24%, respectively. These findings suggest that neoxanthin is effective in decreasing intracellular ROS concentrations, thereby offering protection against oxidative damage [108].

Certain anti-oxidant enzymes, such as heme oxygenase-1 (HO-1) and superoxide dismutase-2 (SOD-2), possess the capability to directly scavenge reactive oxygen species (ROS), thereby decreasing cellular damage induced by oxidative stress [110]. Notably, neoxanthin has been observed to enhance cellular anti-oxidant capacity by upregulating the expression of intracellular anti-oxidant enzymes, specifically HO-1 and SOD-2. Experimental results indicate that H_2_O_2_ treatment significantly diminishes the expression of HO-1 and SOD-2 in HepG2 cells. However, pretreatment with neoxanthin markedly restores the expression levels of these enzymes. Specifically, neoxanthin increases the expression of HO-1 by 17% and 22%, and SOD-2 by 21% and 35% at concentrations of 0.05 μM and 0.1 μM, respectively [108].

Neoxanthin also augments the anti-oxidant defenses of HepG2 cells through the modulation of transcription factor expression [108,111]. Numerous studies have demonstrated that the activation of the nuclear factor erythroid 2-related factor 2/anti-oxidant response element (Nrf2/ARE) pathway leads to an increased expression of anti-oxidant enzymes, which play a crucial role in safeguarding cells against oxidative damage [112]. Experimental evidence indicates that treatment with H_2_O_2_ significantly suppresses Nrf2 expression, whereas pretreatment with neoxanthin at 0.1 μM markedly restores Nrf2 expression by up to 42%. The activation of Nrf2 through neoxanthin pretreatment facilitates the upregulation of downstream anti-oxidant enzyme genes, such as HO-1 and SOD-2, thereby enhancing the cellular anti-oxidant capacity [108].

Oxidative stress is generally associated with the induction of apoptosis [113,114]. Experimental findings indicate that neoxanthin confers cellular protection by inhibiting the apoptotic signaling pathway. In HepG2 cells, treatment with H_2_O_2_ induces oxidative stress, leading to a significant increase (60%) in the expression of the pro-apoptotic protein Bax and a concurrent decrease (50%) in the expression of the anti-apoptotic protein Bcl-2. However, pretreatment with neoxanthin for 3 h effectively mitigates this oxidative stress-induced upregulation of Bax and prevents the reduction in Bcl-2 expression. Notably, the treatment with neoxanthin at 0.1 μM demonstrates the most pronounced reversal effect, restoring approximately 51% and 44% of the expression levels of Bax and Bcl-2, respectively, thereby significantly inhibiting apoptosis induced by oxidative stress [108].

In conclusion, neoxanthin demonstrates its anti-oxidant properties through multiple mechanisms, including the direct scavenging of reactive oxygen species (ROS), preservation of mitochondrial function, activation of endogenous anti-oxidant enzymes, regulation of transcription factor expression, and inhibition of apoptosis [108]. Collectively, these mechanisms render neoxanthin highly effective in safeguarding HepG2 cells against oxidative stress induced by H_2_O_2_ [108].

Collectively, neoxanthin has demonstrated good anti-oxidant activity in various assessment of in vitro anti-oxidant activity [13,108]. Oxidative stress is implicated in the pathogenesis of diverse disorders, including cancer, inflammatory and hyperpigmentation disorders, neurodegenerative diseases, diabetes, and cardiovascular diseases [115]. Thus, this shared biochemical mechanism suggests neoxanthin a promising candidate compound for the prevention and/or treatment of the coexistence of these multiple diseases. Further studies can be carried out to validate the anti-oxidant properties of neoxanthin in multiple anti-oxidant activities (in vivo and in vitro); additionally, the anti-oxidant mechanisms are required to be determined for its further application in the food industry [116].

### 6.3. Anti-Obesity Activity

Presently, the rising prevalence of overweight has become an immense challenge for human health [117,118]. Various carotenoids have exhibited promising anti-obesity effects, including fucoxanthin, lutein, and zeaxanthin [119,120]. Notably, neoxanthin has also shown remarkable prevention effects on fat accumulation in adipocytes [121].

In a study assessing the suppressive effects of 13 natural carotenoids on the adipocyte differentiation of 3T3-L1, treatment with neoxanthin significantly reduces lipid accumulation, as well as glycerol-3-phosphate dehydrogenase activity [122]. Compared to the untreated controls, neoxanthin treatment with 5 μM reduces intracellular lipid accumulation by 32%, while neoxanthin treatment with 20 μM suppresses lipid accumulation to 36% of control levels. The dose-dependent inhibition observed in this study substantiates the capability of neoxanthin to reduce lipid accumulation in adipocytes. Notably, neoxanthin treatment with 20 μM has resulted in a reduction in glycerol-3-phosphate dehydrogenase (GPDH) activity (a key terminal differentiation marker) by around 40% compared to fully differentiated adipocytes, indicating an interference with adipocyte maturation. Additionally, the mRNA expression levels of critical adipogenic transcription factors, C/EBPα and PPARγ, have been significantly downregulated; specifically, 20 μM neoxanthin has reduced C/EBPα expression by 86% and markedly suppressed PPARγ. Given their roles as master regulators of adipogenesis, the coordinated repression of these factors directly decreases differentiation programs. These mechanisms collectively obstruct the differentiation of 3T3-L1 preadipocytes, thereby diminishing adipogenesis and lipid deposition. In addition, the analysis based on the examination of structure and function suggests that neoxanthin has suppressive effects on adipocyte differentiation in 3T3-L1 cells [122].

Fucoxanthinol, a bioactive metabolite of fucoxanthin, is considered the real molecular structure responsible for the anti-obesity effects attributed to fucoxanthin [121,123]. The chemical structures of neoxanthin and fucoxanthinol are very similar, both containing functional groups such as conjugated polyene chains, epoxy groups, and diene groups [20,124]. This molecular similarity implies that neoxanthin may exert its anti-obesity effects through mechanisms similar to those of fucoxanthinol [119]. In particular, the shared structural features present in both compounds are likely to play a crucial role in mediating their biological activity against adiposity [125]. In addition, given the good safety of neoxanthin, it is worthwhile to conduct further research on the weight loss effects and mechanisms of neoxanthin [13].

### 6.4. Anti-Inflammatory Activity

A study has investigated the impact of neoxanthin on inflammation in renal cells, specifically evaluating its effects on inflammatory molecules. The chronic renal failure (CRF) model group has been established by administering a diet containing 0.2% adenine mixed with powdered food to Wistar rats over a period of five weeks. Western Blot analysis reveals that, relative to the control group, the expression levels of tumor necrosis factor-α (TNF-α), interleukin-6 (IL-6), and interleukin-1β (IL-1β) have been markedly elevated in the model group. Conversely, treatment with neoxanthin results in a significant downregulation of TNF-α, IL-6, and IL-1β mRNA expression. These findings suggest that neoxanthin effectively reduces the expression of inflammatory factors TNF-α, IL-6, and IL-1β, while concurrently downregulating the activity of inflammation-associated signaling pathways, including nuclear factor kappa-B(NF-κB). Further preliminary mechanistic analysis indicates that inflammation-related signaling pathways, such as v-rel reticuloendotheliosis viral oncogene homolog A (p65), nuclear factor kappa B subunit 1(p50) have been significantly downregulated following neoxanthin treatment [126].

### 6.5. Anti-Bacterial Activity

An academic study has examined the anti-bacterial effects of neoxanthin on a specific strain of Helicobacter pylori (ATCC43504). The micro-dilution broth method is employed to ascertain the minimum inhibitory concentration (MIC). The findings reveal that the MIC_50_ value for all-trans neoxanthin is 11 μg/mL, indicating potent anti-H. pylori activity. In comparison, the MIC_50_ value for 9′-*cis*-neoxanthin is 27 μg/mL, demonstrating slightly lower activity than (all-trans)-neoxanthin, yet still exhibiting a significant anti-bacterial effect. This study suggests that neoxanthin, particularly (all-trans)-neoxanthin, possesses notable anti-bacterial activity against H. pylori, with its MIC50 value being substantially lower than that of metronidazole. The anti-bacterial efficacy of neoxanthin may be attributed to specific functional groups within its molecular structure, such as monofuranoid rings or allenic bonds, which are likely critical to its anti-bacterial properties. As a carotenoid naturally occurring in plants, neoxanthin’s potential application in anti-bacterial technology merits further investigation. Neoxanthin could potentially serve as an alternative or complementary therapy, offering novel approaches for the treatment of Helicobacter pylori infections [127].

Currently, some studies have been investigated neoxanthin absorption in animal models and human. First, to assess the bioavailability of neoxanthin, a dietary intervention study has been conducted involving the consumption of raw young spinach leaves (100 g per day for four weeks) by 14 participants (mean age 36.5 ± 8.0 years; male–female ratio = 9:5). The concentrations of neoxanthin, neochrome, *β*-carotene, and lutein in both the spinach and the participants’ blood samples (collected pre- and post-intervention) are quantified using HPLC. The results reveal that neither neoxanthin nor neochrome is detectable in the blood samples, while the concentrations of *β*-carotene and lutein exhibited significant increases, by 1.4-fold and 1.9-fold, respectively, over the course of the study. These findings suggest that the bioavailability of neoxanthin in humans is low, indicating that it is unlikely to exert an inhibitory effect on fat accumulation in vivo, contrary to in vitro results. Although consuming raw leafy vegetables may preserve high levels of neoxanthin, it does not enhance its bioavailability [128].

Two epoxyxanthophylls, neoxanthin and fucoxanthin, have been documented to exert antiproliferative effects on various human cancer cell lines in vitro. A study has estimated the intestinal absorption of neoxanthin and fucoxanthin in humans. The plasma concentrations of epoxyxanthophylls before and after a 1-week dietary intervention involving spinach (*Spinacia oleracea*) and wakame (*Undaria pinnatifida*) have been evaluated. The epoxyxanthophylls and their metabolites in plasma extracts are quantified using HPLC following partial purification and concentration via solid-phase extraction cartridges. Despite a 1-week intake of spinach providing 3.0 mg of neoxanthin per day, plasma concentrations of neoxanthin and its metabolites (neochrome stereoisomers) remain exceedingly low (approximately 1 nmol/L), whereas levels of *β*-carotene and lutein show significant increases. Similarly, the plasma concentration of fucoxanthinol, a gastrointestinal metabolite of fucoxanthin, is less than 1 nmol/L after a 1-week intake of wakame providing 6.1 mg of fucoxanthin per day. These findings suggest that the plasma response to dietary epoxyxanthophylls is minimal in humans, even following a 1-week consumption of diets rich in these compounds [27].

These results suggest that the bioavailability of neoxanthin in humans is extremely low. The metabolites of neoxanthin in humans is unclear. Notably, an investigation has been conducted into the metabolites of neoxanthin in mice. The findings indicate that two hours following the oral administration of neoxanthin at a dosage of 40 nmol per mouse, both neoxanthin and (*R*/*S*)-neochrome are detected in the plasma and liver tissues of the mice. The concentrations of neoxanthin, (8*R*)-neochrome, and (8*S*)-neochrome are approximately 13.6, 10.3, and 11.3 nmol/L in the plasma, and 7.3, 10.4, and 6.9 pmol/g in the liver, respectively. Additionally, (*R*/*S*)-neochrome is identified in the small intestinal contents of the mice administered with neoxanthin [129].

Similarly to fucoxanthin, the bioavailability of neoxanthin is low. These results may to attribute to the similarity of chemical structures of the two compounds. Currently, the metabolites of neoxanthin are not yet well characterized. Hence, more studies are required to develop the metabolites of neoxanthin or advance neoxanthin as a drug precursor to be further activated by in vivo metabolism.

## 7. Current Challenges and Opportunities

Compared with commercial carotenoids (e.g., astaxanthin, lutein and fucoxanthin), neoxanthin has not yet been industrially produced. Considering the several health benefits of neoxanthin in human health, it is a promising carotenoid for further investigation. To meet the demand of neoxanthin production in commercial scale, some major challenges have been faced, and future research is expected to focus on some aspects as follows.

### 7.1. The Prospect of Microalgae as a Neoxanthin Source

Compared to high plants, microalgae are regarded as promising neoxanthin sources due to some main advantages including higher neoxanthin content, multiple species of microalgae, higher growth rate, and immune to seasonal variations [130]. The content of neoxanthin in the microalgae is in the range of 0.26~30,880 μg/g dry weight [11,12]. Notably, the neoxanthin content in the dry powders of *Chlorella vulgaris* is 30.88 mg/g [11]. The complex and limited genetic potential of microalgae constrains pigment production, presenting a significant challenge to their commercial utilization. To overcome the obstacle, the application of genetic and metabolic engineering techniques is regarded to be an effective strategy. Metabolic engineering and synthetic biology strategies have been used to enhance microalgal pigment production, including lutein; *β*-carotene, fucoxanthin, zeaxanthin and astaxanthin [131]. In addition, an effective and economic photoautotrophic cultivation systems is vital for microalgal neoxanthin production. The future research is expected to focus on some aspects including the suitable photobioreactors, optimal light supply, temperature, effective capture of carbon dioxide, and protection of the cultures from contamination [132]. Currently, tubular photobioreactors are the most common industrial photobioreactors, which has been applied for the production of astaxanthin [133].

### 7.2. Extraction and Purification of Neoxanthin

Some progress has been made for the extraction of neoxanthin from high plants or microalgae biomass, including organic solvents extraction [42,53], ionic liquids extraction [54,55], supercritical liquid extraction [56], ultrasound-assisted extraction [60,61], and pressurized liquid extraction [11]. Considering the high-polarity of neoxanthin, the use of polar organic solvents including dimethyl ether, acetone, and ethanol, can enhance the extraction efficiency [42]. In addition, ionic liquids technology has been investigated for the extraction of neoxanthin by using some mixture composed of ionic liquids and/or organic solvents [54]. Notably, the choice of a suitable extraction solvent remains a key challenge for sustainable extraction processes in a large-scale. A truly optimal solvent is fully enabled to meet the safety requirement of neoxanthin in the foods and cosmetics fields and facilitate the further purification of neoxanthin. Generally, ethanol is regarded as an optimal solvent due to its green, high-polarity, and safety.

To the best of our knowledge, the purification technology of neoxanthin has not yet been found. The purification of neoxanthin remains a great challenge due to the lack of suitable raw material with high content of neoxanthin, the co-existed compounds with similar polarity, the chemical instability of neoxanthin, the lack of purification technology of neoxanthin with the property of green, efficient and economic feasibility. The development of purification technology of neoxanthin is an essential research aspect for the further application of neoxanthin. The establishment of purification technology can provide necessary high-purity neoxanthin for the further studies of biological activity of neoxanthin. Some xanthophyll carotenoids (such as lutein, fucoxanthin) have been commercial produced. The successful purification of these xanthophyll carotenoids can provide good cases for the further purification of neoxanthin.

Several advanced purification techniques have been documented, including the selective adsorption of lutein on a solid phase [130], as well as reversed-phase HPLC and high-speed counter-current chromatography for obtaining small quantities of high-purity carotenoids [134,135]. Additionally, supercritical anti-solvent precipitation has been shown to produce solid carotenoids within a matter of minutes [136].

### 7.3. Biological Activity of Neoxanthin

Carotenoids are a class of compounds with diverse bioactivities including anti-cancer [89], anti-oxidant [11], anti-obesity [119], anti-inflammatory [126,137], anti-senile dementia as well as ocular-protective [138]. Presently, neoxanthin exhibits some activities such as anti-cancer, anti-oxidative, and anti-obesity activities [13]. Some new technology such as high throughput screening, can facilitate the efficient studies of neoxanthin. Recent investigations have explored the absorption of neoxanthin in both animal models and humans. Similarly to fucoxanthin, neoxanthin exhibits low bioavailability, which may be attributed to the structural similarities between the two compounds. Currently, the metabolites of neoxanthin remain inadequately characterized. It is known that dietary neoxanthin is partially converted into (*R*/*S*)-neochrome due to intragastric acidity before intestinal absorption. Notably, (*R*/*S*)-neochrome demonstrates an antiproliferative effect on PC-3 cells without significantly inducing apoptosis. The limited bioavailability of neoxanthin presents a significant challenge for its clinical application. Fortunately, strategies such as combining fucoxanthin with dietary oil or milk, or incorporating it into nanoparticles, have been developed to enhance its bioavailability [139]. These successful cases can provide practical and feasible solutions for improving the bioavailability of neoxanthin.

## 8. Conclusions

Carotenoids are a promising class of compounds with diverse beneficial effects on human health. Some carotenoids such as lutein, fucoxanthin, and astaxanthin, have been produced on a commercial scale; in additionally, the global carotenoids market has experienced rapid expansion in recent years. In this review, we systematically reviewed the current progress on the structure, source, biosynthetic pathways, extraction methods and bioactivities of neoxanthin. Moreover, this review discussed the major challenges of neoxanthin production from microalgae, and proposed some future development prospects and potential solutions. Overall, this review provided useful information in the future research aspects of neoxanthin.

## Figures and Tables

**Table 4:** Methods for the extraction of neoxanthin.

Extraction Methods	Plant Species	Extraction Solvents	Extraction Conditions	Neoxanthin Yield	Reference
Organic solvents extraction	*Chlorococcum humicola*	Dimethyl ether	45:1 Solid/liquid ratio (*w*/*w*), 41 °C, for 20 min	2.55 mg/g	[53]
	*Caryocar Brasiliense*	Acetone	3 mL/g Solid/liquid ratio, for 300 s, three times extraction	0.70 μg/g	[55]
	*Scenedesmus obliquus*	Ethanol	10 mL/g Solid/liquid ratio, for 3 min, three times extraction	180.33 μg/g	[42]
	*Brazil Pouteria*	Acetone	40–60 °C	193 μg/g	[55]
Ionic liquids extraction	*Caryocar Brasiliense*	1:3 (1-hexyl-3-methylimidazolium chlorid): ethanol (*v*/*v*)	3 mL/g Solid/liquid ratio, for 300 s, three times extraction	1.88 μg/g	[55]
	*Chlorella sorokiniana*	1:4 (1-hexyl-3-methylimidazolium chloride): ethanol (*v*/*v*)	10 mL/g Solid/liquid ratio, for 7.5 min, two times extraction	0.03 mg/g	[54]
	*Scenedesmus obliquus*	1:3 (1-butyl-3-methylimidazolium tetrafluoroborate): ethanol (*v*/*v*)	10 mL/g Solid/liquid ratio, for 3 min, three times extraction	48.71 μg/g	[42]
	*Scenedesmus obliquus*	1:3 (1-butyl-3-methylimidazolium tetrafluoroborate): ethanol (*v*/*v*)	10 mL/g Solid/liquid ratio, for 3 min, three times extraction	122.66 μg/g	[42]
Supercritical liquid extraction	*Scenedesmus* sp.	9:1 CO_2_: Ethanol (*v*/*v*)	Pressure 300 bar, 60 °C, and CO_2_ flow rate 2 mL/min, for 60 min	670.8 μg/g	[56]
Ultrasound-assisted extraction	*Cucurbita moschata*	2:1 Ethanol: Petroleum (*v*/*v*)	31 mL/g solid/liquid ratio, 203 W, for 30 min	36.69 μg/g	[58]
	*Chlorella vulgaris*	Ethanol	30 mL/g Solid/liquid ratio, amplitude wave of 20%, 25 °C, for 25 min	9.83 mg/g	[11]
Pressurized liquid extraction	*Chlorella vulgaris*	Ethanol	Pressure 1500 psi, 50 °C, for 20 min	11.35 mg/g	[11]

**Table 5:** The multi-target mechanism of action of neoxanthin in multiple cancer cell lines.

No.	Type of Cancer	Cell Line	Target	Mechanism of Action	Reference
1	Colon cancer	HCT116	mitochondrial function	Caspase-independent apoptotic pathway via loss of mitochondrial transmembrane potential, involving apoptosis-inducing factor (AIF), cytochrome-C, and endonuclease G (EndoG)	[93]
2	Prostate cancer	PC-3	Caspase-3	Caspase-3-dependent apoptosis	[94]
3	Gastric cancer	molecular docking	Cytotoxin-associated gene A (Cag-A)	Inhibits the binding of Cag-A of Helicobacter pylori to phosphatidylserine on host cell membranes	[95]
4	Lung cancer	A549	Caspase-3	Induce apoptosis through caspase-3 activation; increase ROS clearance and repair activity with IC50 (mg/L) = 7.5 ± 0.6 μM	[96]
5	Cervical cancer	HeLa	Caspase-3	Modulation of the activity of various transcription factors and responsive elements; Inhibition of the clonal expansion of initiated cells through enhanced gap junctional communication; Immunomodulatory effects by enhancing tumor immunity with IC50 (mg/L) = 3.8 ± 0.2 μM	[96]

**Table 6:** Anti-oxidant mechanisms of neoxanthin in H_2_O_2_-treated HepG2 cell model.

No.	Mechanism of Action	Experimental Data Results	Reference
1	Activating endogenous anti-oxidant signaling pathways	At 4 h exposure to 0.5 mM H_2_O_2_, the cell viability in the neoxanthin pretreatment groups (0.05 μM and 0.1 μM) is significantly greater compared to the group subjected solely to H_2_O_2_ treatment	[108]
2	Directly removes reactive oxygen species (ROS) produced within cells	Treatment of HepG2 cells with H_2_O_2_ resulted in a 38% increase in reactive oxygen species (ROS) levels compared to the control group. Pretreatment with neoxanthin at concentrations of 0.05 μM and 0.1 μM resulted in a 12% and 24% reduction in ROS production	[108]
3	Upregulates the expression of intracellular anti-oxidant enzymes (HO-1 and SOD-2)	H_2_O_2_ treatment significantly diminishes the expression of HO-1 and SOD-2, neoxanthin increases the expression of HO-1 by 17% and 22%, and SOD-2 by 21% and 35% at concentrations of 0.05 μM and 0.1 μM	[108]
4	Regulates transcription factor (Nrf2/ARE) expression	Treatment with H_2_O_2_ significantly suppresses Nrf2 expression, whereas pretreatment with neoxanthin at 0.1 μM markedly restores Nrf2 expression by up to 42%	[108]
5	Inhibits the apoptotic signaling pathway	Treatment with H_2_O_2_ leading to a significant increase (60%) in the expression of the pro-apoptotic protein Bax and a concurrent decrease (50%) in the expression of the anti-apoptotic protein Bcl-2. Treatment with neoxanthin at 0.1 μM demonstrates the most pronounced reversal effect, restoring approximately 51% and 44% of the expression levels of Bax and Bcl-2	[108]

## Data Availability

The data presented in this study are available on request from the corresponding author.
